# Notes and News

**Published:** 1887-02-12

**Authors:** 


### Notes and News.

Collected and Communicated.

Lord Wolverton has just made the magnificent donation of £1,000 for the institution of a "Wolverton Room" at Morley House, the convalescent home established in connection with the Hospital Saturday Fund. The room, which is to contain four beds, is to be entirely devoted to the use of London postmen.

A piece of land adjoining the Bedford Infirmary is about to be bought by the Governors of the Duke of Bedford's trustees, for the sum of £3,350. Mr. Samuel Whitbread, M.P., has made a donation of £1,000 to the building fund of the new infirmary, and a similar sum has been promised by the Duke of Bedford.

Messrs. Louis Cohen & Sons, and Messrs. Bristowe Brothers, recently started a collection among the members of the Stock Exchange in aid of the fund now being raised in support of Guy's Hospital. No less than twenty-four firms contributed a hundred guineas apiece. Altogether a sum of £5,730 was collected and paid over to the Mansion House Fund.

Mrs. Bancroft, the popular actress, has very kindly promised to recite, on the 18th and 19th inst., at the Novelty Theatre, in aid of the funds of the Belgrave Hospital for Children, Gloucester Street, S.W.

The inhabitants of Folkestone have decided to celebrate the Queen's Jubilee by the erection of a large hospital. A generous lady forwarded the Mayor a cheque for £1,000. Several persons have subscribed £100 each. We are sorry to hear from Canterbury that the Jubilee scheme for endowing a ward in the County Hospital has been found impracticable. Something like £10,000 would have been required for this purpose, a sum which it seems quite impossible to raise locally.

Buxton, which so loyally celebrated the Queen's accession fifty years ago, has not made up its mind as to the mode in which it will locally crown the Jubilee. A Cottage Hospital has been suggested; but surely the renowned Devonshire Hospital and Buxton Bath Charity should be able to attend to the injuries and sicknesses of the townsfolk, as well as to its rheumatic visitors.

Thieves recently broke into the Dolphin Hotel, Faversham, and among the booty with which they succeeded in eloping was a contribution box of the Kent and Canterbury Hospital. The amount of the cash which it chanced to contain is of course unknown.

The need which exists for such a publication as The Hospital, and its great possibilities of usefulness and help to the public, have been lately demonstrated in a manner which cannot but prove highly gratifying to its promoters. Mr. R. C. Mossman lately wrote to us from Edinburgh, desiring certain precise information about hospitals, which an exhaustive "search through all the books on the subject contained in the Advocates' Library" had failed to obtain. "As a last resource" he looked through a file of *The Times*, and found the letter of January 5th signed by the editor of this paper. This induced him to apply to us for the information required, and we were able to refer him to the proper source, and so to relieve him of any further trouble in the matter. Mr. Mossman has written us a grateful letter of acknowledgment, and we mention the circumstances just detailed, not from any spirit of self-glorification, but that we may have an opportunity of assuring our readers that we shall always be glad to give them every possible help in similar difficulties, if it lies in our power to do so.

Very considerable diversity of opinion appears to exist among the residents of Blackheath and the neighbourhood, with respect to the erection of a Jubilee Hospital. The Blackheath and Charlton Cottage Hospital is at present quite insufficient to provide for the needs of a population of over 50,000 persons, but its friends are opposed to its break-up and its supersession by a big hospital. The proposal for the erection of a new building has been referred to the Hospital Committee, and a general meeting will shortly be called.

Few hospitals can boast of being in the happy condition, financially speaking, of the Royal Free. A few weeks since we gave a sketch of the eventful history of this institution, which was founded by the late Dr. William Marsden. After a career of fifty-nine years, chequered at one time by many troubles, both from within and without, the Royal Free is now in a prosperous condition. Last year no less a sum than £15,341 12*s.* 4*d.* was received from legacies, five times the amount from this source received in 1885. This good fortune has enabled the committee to invest £6,000 in the purchase of Consols, thus raising the invested capital of the hospital to £20,704 6*s.* 10*d.* Last year 1,813 in-patients were treated at the Royal Free Hospital, while the out-patients who received advice and medicine numbered 22,191.

Southend has determined to found a Victoria Hospital, as a suitable memorial of the approaching Jubilee. No town has, perhaps, made greater strides in improvement in recent years than Southend. A hospital is now, practically speaking, its only want, and this the inhabitants mean shortly to supply. The total cost of the hospital, which is to have eight beds to start with, will be about £2,500. The permanent cost of keeping up the hospital is estimated at £250 a year, a sum which it is proposed to meet by donations, subscriptions, and the establishment of Hospital Saturday and Sunday Funds.

THE following vacancies are announced this week, the amount of the salary being placed in each case after the name of the institution :—

**Honorary Medical Officer.**

Royal Albert Hospital, Devonport. Surgeon, doubly qualified.

**Resident Medical Officers.**

Doncaster Infirmary. House Surgeon, doubly qualified. —£100.
Tunbridge Wells Hospital. House Surgeon, doubly qualified. —£100.

**Matron.**

Surrey County Hospital, Guildford.—£65, with board, etc.

**Trained Nurses.**

Carlisle Infirmary. Night.—£24, and uniform.
Grimsby Hospital. One.—£20, and uniform.
Hertford Infirmary. One assistant.—£16 to £20.
Nottingham Nursing Institution. Several.

**Probationers.**

Hertford Infirmary.—£12.
Norwich Children's Hospital. Lady.—£1 per week.

---

THE sixth entertainment for the benefit of the in-patients of the Cancer Hospital, Brompton, was given in the out-patients' room at the hospital on the 3rd inst. The programme was principally dramatic, interspersed with music and singing, and consisted of F. W. Broughton's comedy, *Written in Sand*, followed by J. B. Buckstone's comic drama, *Good for Nothing*. All the parts were thoroughly well sustained, but the palm of the evening must be awarded to Miss Mellon for the excellence of her acting, particularly in the character of "Nan," in the last piece. Upwards of seventy of the patients attended and it was evident that they thoroughly appreciated the kindness of the several ladies and gentlemen who had contributed to their amusement. Dr. Marsden, Mr. Carr, Rev. N. Liberty, Mr. Jessett, and other visitors were present.

---

THE Jubilee arrangements for increasing the contributions to the London hospitals on Hospital Sunday, on the 20th June next, are exciting a good deal of interest. A meeting will be held at the Mansion House on Tuesday, June 14th, at 2.30 o'clock, at which the Lord Mayor will preside. Lord Salisbury has consented to attend and move the first resolution, and Canon Fleming is working with his unequalled energy and enthusiasm to make this meeting a grand success. The Prime Minister's example is likely to prove contagious, and there is the best of reasons for hoping that the Hospitals Week and Hospital Sunday in this year of Jubilee will be worthy of the greatest city of the Empire.

---

A NEW Hospital for Consumption and Diseases of the Chest and Throat is to be built at St. Leonards, on an excellent site on the West Hill. The inception of this scheme is due to the Committee of the Friedenfels Home, a little institution, situate at Upper Maze Hill, St. Leonards, which was established in October 1884, for the treatment of pulmonary diseases. During the first twelve months the Home admitted eighty-two patients, sixty-five of whom came from London; last year it received 101, the metropolis contributing ninety. The Home has now seventeen beds, always full with poor patients mostly in the second and third stages of consumption. The unavoidable rejection of so many deserving cases has decided the committee to enlarge their sphere of action in the direction we have indicated. The air of St. Leonards is very mild and balmy, and the proposed site, which is within easy distance of London, is admirably suited for its intended purpose. In all large centres of population, and in the metropolis especially, consumption now plays fearful havoc. It is estimated that there are not less than 80,000 persons in England annually suffering from this disease, while in London its death-roll is computed at from 8,000 to 9,000 per annum. At present the provision for the treatment of such cases is sadly deficient. Moreover, at some of these, patients are only admitted when the disease is in its earlier stages, and relief or cure can safely be anticipated from treatment. Dr. Hermann Weber, in the Croonian Lectures for 1885, speaking of the special hospitals for consumption, expressed his opinion that if all the beds were added together, the provision would be almost infinitesimal compared with the number of consumptive patients belonging to the poorer classes. Dr. Schneer's recent proposal to establish a consumption hospital on the Riviera for the treatment of the poor is excellent in principle, but wanting in practicability in all ordinary cases. Patients of the poorer classes who are the victims of this disease must find treatment nearer home. A hospital such as the Committee of the Friedenfels Home propose to erect at St. Leonards, so accessible from the metropolis, would be one of the best memorials of our Queen's Jubilee which Englishmen could devise. The plans of the new building are now in course of preparation. It is proposed to arrange the ordinary wards for the reception of about fifty patients, of both sexes, while private wards will be added for sufferers desiring special attention or privacy. A Council and an Executive Committee are now being formed to carry out the projected undertaking, and an office has been opened at 93, Fleet Street, where all information can be obtained from the secretary.

---

AT Windsor Royal Infirmary, on January 14th, an entertainment, got up entirely through the exertions of the resident officers of that establishment, was given to the ward patients. Invitations were also issued to those living near, who had enjoyed the benefits of this institution during the past year, to which about seventy responded. In order to prevent any stiffness or formality on the occasion, no ladies or gentlemen were asked to be present, except those who assisted in the entertainment, and a thoroughly bright social spirit reigned throughout the evening. The convalescent ward which seated the audience had been prettily decorated by the in-patients, and a small stage erected at one end. On this the farcical play, called *A Silent Woman*, was performed with much spirit, to the hearty amusement of the spectators, who also testified their approval of the remainder of the programme, which consisted of music, songs, recitations, and a hornpipe danced by a former patient. In the course of the evening refreshments were liberally provided, with the additional fun of costume-crackers, highly appreciated by young and old. After "God Save the Queen" had been sung, tea and coffee were served in the waitingroom, and the party finally broke up, with many expressions of gratitude and pleasure from those who had thus been privileged to renew the kindly feeling to which their previous acquaintance with the Royal Infirmary had given rise.

---

THE following sums have been recently received by the various Medical Charities, the name of the donor or the source from which they were obtained being given in each instance after the name of the Institution :—

**Legacies.**

Isle of Wight Infirmary, Ryde. Mrs. Harriet Beckford, £1,500
Hospital for Incurables, Putney. Mrs. Harriet Beckford, £1,000.
Metropolitan Convalescent Institution, Walton-on-Thames. Mrs. Harriet Beckford, £500.
National Hospital for Consumption, Ventnor. "Delta," £10 10*s.*
Dental Hospital of London, Leicester Square. Sir Charles McGrigor, £20.

**Donations.**

North London Hospital. Mr. L. W. Longstaff, £35. Mr. Richard Moon, £10. Mr. Hugh Martineau, £50.
Home for Incurable Children, Maida Vale. Mr. E. B. Randall, 10 guineas.
West End Hospital for Nervous Disorders, Welbeck Street. Messrs. Barron, Harvey, & Co., £10 6*s.* 10*d.*
Charing Cross Hospital. Mrs. Peto, 10 guineas.
North Eastern Hospital for Children. Mrs. Harrison, £25.

**Subscriptions.**

Dental Hospital of London, Leicester Square. Mr. H. A. Brassey, 10 guineas.

---

A KENSINGTON correspondent, who signs himself "F.," sends us yet another proposal for a Jubilee memorial. This is what he says :—"As a consequence of reading Archdeacon Farrar's excellent paper dealing with some of the main causes of poverty, it has suggested itself to me that the lack of self-help and the thriftlessness amongst our poorer classes is engendered in a large measure by ignorance and lack of opportunity. It seems to me that no more fitting memorial of our Queen's Jubilee Year could be instituted than a society having for its object the placing within the means and the knowledge of our toilers of the best modes of self-help. Such a society I would call the Victoria Society, and in addition to a provident fund, savings-bank, etc., directly tending to thrift, I would have cheap cooking classes for women, and a labour bureau, with lectures, for the men. The society is doubtless wide, but so is the evil to be cured."

---

DR. SYDNEY PHILLIPS delivered an interesting lecture to the members of the Midwives' Institute and Trained Nurses' Club on January 21st, at the Rooms of the Institute, 15, Buckingham Street, Strand. The subject of the lecture was "The Diseases of Infants and Children," and Dr. Phillips drew special attention to the need of close observation on the part of nurses, and in those cases in which it was difficult for the diagnosis to be arrived at by the medical man. The various cries of a young infant can be differentiated quickly by an experienced ear, and the expression and furrows on the face as the marked ellipsis in stomachic pain, the movement of the limbs, and the attitudes of the child when sleeping, are characteristic of different degrees and kinds of pain. The pulse, the respirations, and the state of the fontanel should also be studied carefully both in health and in disease, and Dr. Phillips pointed out these as frequently the only tests of illness in young children, as in some cases *pain* was not complained of, and, as in pleurisy, the inflammatory condition might be far advanced before its gravity was discovered. In cases of croup, Dr. Phillips recommended that while the hot bath was being prepared cold water should be thrown over the head and throat of the child, this being the best means of producing the needful "full respiration"—in fact, the cold douche he considers a necessary adjunct to the hot bath in cases of convulsions and of croup. Attention was drawn to the need of careful observation in children with choreic tendencies ; often the first attacks were heralded by exceeding slowness and seemingly wilful stupidity—things were dropped and broken, and the child itself occasionally stumbled and fell, through seeming inattention. Nurses were urged to be careful not to recommend punishment in such cases, as nervous terror and sharp physical pain frequently hastened the attacks. The importance of diet was dwelt on, and the lecturer pointed out that a disordered digestion was a far more frequent factor in the production of alarming symptoms in young children than was generally supposed ; the rapidity with which they appeared, or disappeared if rightly treated, was a matter both of alarm and of thankfulness to the attendants. A vote of thanks to the lecturer was proposed by Mrs. Humphries and was seconded by Miss Frennan, Matron of the British Lying-in Hospital.

---

MR. GEORGE C. ROWLAND, the secretary of the Richmond Hospital, has sent us a list of box collections made in 1886, in aid of the funds of that institution. The income derived from this source has materially increased during the past year, and a great and most welcome feature of this increase is the liberal support given by the working men of the neighbourhood, which is evidenced by the long list of sums, varying in amounts, obtained from the boxes placed in the different workshops. The total amount collected by means of boxes last year was £280 3*s.* 6*d.*, which pleasing fact testifies to the local appreciation of the charity, and the self-denial of many of the inhabitants. Accidents are received at all times without any letter of recommendation being required, and as this is now widely known, cases from all parts of the surrounding parishes are brought at all hours of the day and night for treatment at Richmond. The total number of in-patients during 1886 was 345, out-patients 2,751, whilst the attendances of out-patients reached the total of 13,054.

---

THE election of Dr. Costine to the position of consulting physician, and of Mr. E. M. Sheldon to that of consulting surgeon to the Stanley Hospital, Liverpool, caused two vacancies on the honorary staff. Dr. Whitford, who has for some time acted as assistant physician, has been appointed to the honorary physicianship ; and Mr. Robert Jones, M.R.C.S., who has also been associated with the honorary staff of the hospital, has been elected honorary surgeon. Mr. Costine and Mr. Sheldon, who were the founders of the Stanley Hospital, have been on the staff of the institution for twenty years.

